# The Predictive Nature of Pseudoneglect for Visual Neglect: Evidence from Parietal Theta Burst Stimulation

**DOI:** 10.1371/journal.pone.0065851

**Published:** 2013-06-18

**Authors:** Alice Varnava, Martynas Dervinis, Christopher D. Chambers

**Affiliations:** 1 Department of Psychology, Swansea University, Swansea, United Kingdom; 2 School of Psychology, Cardiff University, Cardiff, United Kingdom; French National Centre for Scientific Research, France

## Abstract

Following parietal damage most patients with visual neglect bisect horizontal lines significantly away from the true centre. Neurologically intact individuals also misbisect lines; a phenomenon referred to as ‘pseudoneglect’. In this study we examined the relationship between neglect and pseudoneglect by testing how patterns of pre-existing visuospatial asymmetry predict asymmetry caused by parietal interference. Twenty-four participants completed line bisection and Landmark tasks before receiving continuous theta burst stimulation to the left or right angular gyrus. Results showed that a pre-existing pattern of left pseudoneglect (i.e. right bias), but not right pseudoneglect, predicts left neglect-like behaviour during line bisection following right parietal cTBS. This correlation is consistent with the view that neglect and pseudoneglect arise via a common or linked neural mechanism.

## Introduction

Manual line bisection (LB), and non-manual perceptual variants of the task (i.e. Landmark task, LM), are amongst the most frequently employed instruments used to diagnose and quantify visuospatial neglect. Following parietal damage, most patients with visual neglect bisect horizontal lines significantly away from the centre and on the ipsilesional side, as if they ignore the contralesional side of the stimulus or are hyper-attentive to the ipsilesional side [1], [2], [3], [4]. The majority of neurologically intact individuals also reliably misbisect lines, although the magnitude of bisection error is much smaller than in neglect patients: a phenomenon commonly referred to as *pseudoneglect* [5]. Neglect and pseudoneglect are often discussed as expressions of a common underlying asymmetry and are assumed to possess a theoretical and neurological relationship to each other [6], [7], [8]. However, although LB errors have repeatedly been studied in both healthy individuals and brain damaged patients, we are not aware of any studies that have directly considered how pre-existing patterns of asymmetry (i.e. pseudoneglect) are related to clinical patterns of neglect.

Many studies have already shown that the two phenomena possess similar susceptibilities to a variety of modulating factors, thus reinforcing the view that they are closely related [7]. For example, both the magnitude and direction of bisection errors in pseudoneglect are modulated by stimulus or task factors (e.g. line length, line location, task instructions: [7], [9]) that similarly influence the magnitude and direction of visual neglect [10], [11]. These reports clearly reflect the asymmetry that defines both the clinical presentation of visual neglect and pseudoneglect. Indeed, most theoretical models of neglect are grounded in the concept of a specific right hemisphere specialisation for spatial processing, just as aphasia reflects left hemisphere specialisation for language processing in the majority of people [6], [12]. This is not surprising given that visual neglect is more frequent, long lasting and severe after right than left hemisphere damage [13], [14]. Furthermore, the majority of pseudoneglect studies report an overall (mean) leftward bisection deviation [15]. These asymmetries have led to several hypotheses concerning the asymmetric contralateral control of visuospatial abilities including attention, representation, midpoint computation and motor intention [16], [17], [18], [19].

Support for a neural link between neglect and pseudoneglect stems from convergent evidence using functional or structural brain imaging, neurodisruption techniques (e.g. transcranial magnetic stimulation, TMS) and lesion studies in patients with neglect (e.g. [20], [21], [22], [23], [24], [25], [26], [27], [28], [29], [30], [31]). Among the most notable is a study by Mort and colleagues [31] in which high resolution MRI was used to map the lesions of 14 right hemisphere patients who had suffered middle cerebral artery stroke. The authors found that the critical area involved in neglect was the angular gyrus of the right inferior parietal lobule.

More recently, Oliveri and Vallar [26] tested 10 neurologically unimpaired participants performing a LM task during delivery of repetitive transcranial magnetic stimulation (rTMS). Stimulation of the right inferior parietal lobule induced a rightward error, symptomatic of left neglect. Using voxel-based lesion-symptom mapping in 80 patients following right hemisphere stroke, Verdon and colleagues [29] further reported that damage to the right inferior parietal lobule near the supramarginal gyrus (with an extension into posterior white matter) was associated with impaired performance on tasks including LB. Most recently, Thiebaut de Schotten et al [28] reported evidence for a larger parieto-frontal network in the right than left hemisphere in 20 healthy participants, and a significant correlation between the degree of anatomical lateralization and asymmetry of their performance on LB.

One concern, however, lies with drawing consistent conclusions from the variety of bisection tasks used to measure neglect and pseudoneglect, with some studies using LB and others LM. Several studies have drawn a distinction between perceptual and perceptual-motor components of line bisection, with some patients demonstrating neglect on manual LB but not on the non-manual LM task, while others show the reverse dissociation [32], [33]. This perceptual/motor distinction is therefore likely to be important for defining more accurately the anatomical correlates of neglect and pseudoneglect [34], [35].

Furthermore, there is considerable variability and inconsistency of LB behaviour in neglect patients and healthy individuals, suggesting that a more complex explanation is required. For example, although visual neglect is more frequent following right than left hemisphere damage, right neglect (i.e. significant left bisection error) is often found in patients with left hemisphere damage [36] suggesting that visuospatial abilities are a bilateral function with right hemisphere dominance in most (but not all) individuals [28], [37]. Also, while many patients with parietal damage demonstrate impaired LB performance, others bisect lines within normal limits [38] but are impaired on other tests of neglect (e.g. cancellation tasks: see [39]), perhaps suggesting individual differences in neural functioning, strategy use or neuroanatomical damage [4], [9], [27], [28].

With regards to healthy asymmetries, left (rather than right) LB errors have been adopted as the definition of pseudoneglect, presumably because leftward errors are directionally opposite to those made most often by patients with visual neglect [8]. However, in a review of the pseudoneglect literature, Jewell and McCourt [3] established that while the mean performance of healthy individuals represents a bisection significantly to the left of true centre (right pseudoneglect), several studies report individuals with reliable rightward deviations (left pseudoneglect) or no significant deviation from centre [40], [41], [42]. This apparent lack of reliability in group studies is perhaps a reflection of individual differences and genuine subtypes of pseudoneglect [15], [40], [43]. Yet most pseudoneglect studies fail to differentiate those individuals who place bisection marks consistently to the left with those that place their mark to the right: potentially a significant confounding factor in many bisection studies since the two may cancel each other out. These observations, collectively, have received little comment despite their theoretical relevance to an understanding of visual neglect and healthy visuospatial processing.

In the present study, we examined the relationship between pseudoneglect and visual neglect by testing how patterns of pre-existing (normal) visuospatial asymmetry predict or inform patterns of pseudo-pathological asymmetry on the manual LB and non-manual LM task. Using continuous theta burst stimulation (cTBS) we sought to apply a direct test of the predictive nature of pseudoneglect for patterns of neglect by transiently disrupting cortical function in healthy left and right posterior parietal regions. Our results show that having an existing pattern of left pseudoneglect that resembles actual left neglect predisposes individuals toward left neglect-like behaviour after right parietal cTBS. Hence, the direction of pre-existing visuospatial asymmetry predicts the behavioural effects of disturbing the parietal cortex.

## Materials and Methods

### 1. Participants

Twenty four volunteers (12 male; 12 female; aged 19–30, 23.7±3.7, mean ± SD) took part in the present study. All were deemed right-handed according to the Briggs and Nebes Handedness Questionnaire [44] and had normal or corrected to normal visual acuity. Prior to testing, participants were screened for contraindications to magnetic resonance imaging (MRI) and TMS [45] and provided written informed consent. The experimental protocol was approved by the School of Psychology Ethics Committee at Cardiff University.

### 2. TMS Protocol

Cortical stimulation was delivered via a biphasic MagStim Rapid2 System using a 70 mm figure-eight induction coil. Prior to testing, structural T1-weighted magnetic resonance (MR) scans were acquired for each participant using a 3T GE HDx scanner (1×1×1 mm, sagittal acquisition). TMS/MR registration was performed using a magnetic tracking device (miniBIRD 500, Ascension Tech) and MRIcro/MRIreg interface software [46]. All TMS parameters were within the international safety guidelines of Rossi et al., [47].

Appropriate levels of stimulation intensity were determined according to a measure of cortical excitability known as distance-adjusted motor threshold (MT). As in previous studies, MTs for left and right M1 were obtained using the ‘observation of movement’ method [48], [49], [50], where MT is defined as the minimum stimulator output required to induce a visible twitch in the contralateral hand on 5 of 10 consecutive pulses, delivered at a rate of ≤1 Hz to the motor cortex. The distance between the scalp and stimulating coil were then manipulated using 0 mm, 6 mm, and 9 mm thick acrylic separators placed between the coil and the scalp surface, resulting in MT measures at four scalp-coil distances and providing a direct index of cortical excitability. Distance-adjusted MTs for left and right M1 were independently determined on different days to ensure that continuous theta burst stimulation (cTBS) was delivered at the same effective intensity. MTs were then used to calculate the required intensities of cTBS at the stimulation site.

In experimental sessions, cTBS was delivered at 80% of distance-adjusted MT for 40 s (600 pulses). Continuous trains of pulses were delivered in triplets of 50 Hz (20 ms between pulses) with a burst frequency of 5 Hz (200 ms between bursts). This protocol can depress cortical excitability in the stimulated area for up to and beyond 60 minutes [51], [52]. During stimulation, the coil was held constant with the handle pointing laterally in a slightly anterior orientation, tangentially to the surface of the scalp.

Sham cTBS was provided over the left or right stimulation site (counterbalanced across participants) with the lateral edge of the figure-eight coil held perpendicular to the scalp. This form of sham stimulation does not produce measurable evoked potentials or changes in regional cerebral blood flow (rCBF) when applied over the motor cortex [53], [54] and serves to reduce perceived changes in stimulator sounds between sham and cTBS without directly stimulating the cortex. An acrylic plastic separator (9.03 mm thick, 200 mm x 100 mm) was positioned flat between the coil and scalp to provide a cautionary barrier between the coil and scalp and to mimic the tactile sensation of a tangentially oriented coil.

### 3. Localisation of Stimulation Site

The location of the stimulation site was guided by three recent studies [26], [29], [31] that describe the neuroanatomy associated with left visuospatial neglect. The presence of left neglect was based on clinical assessment including performance on the LB or LM task. The Montreal Neurological Institute (MNI) coordinates for the regions associated with neglect in the three studies were: 46, −44, 29 [31]; 64, −40, 52 [26]; and 33, −47, 37 [29].

**Figure 1:**
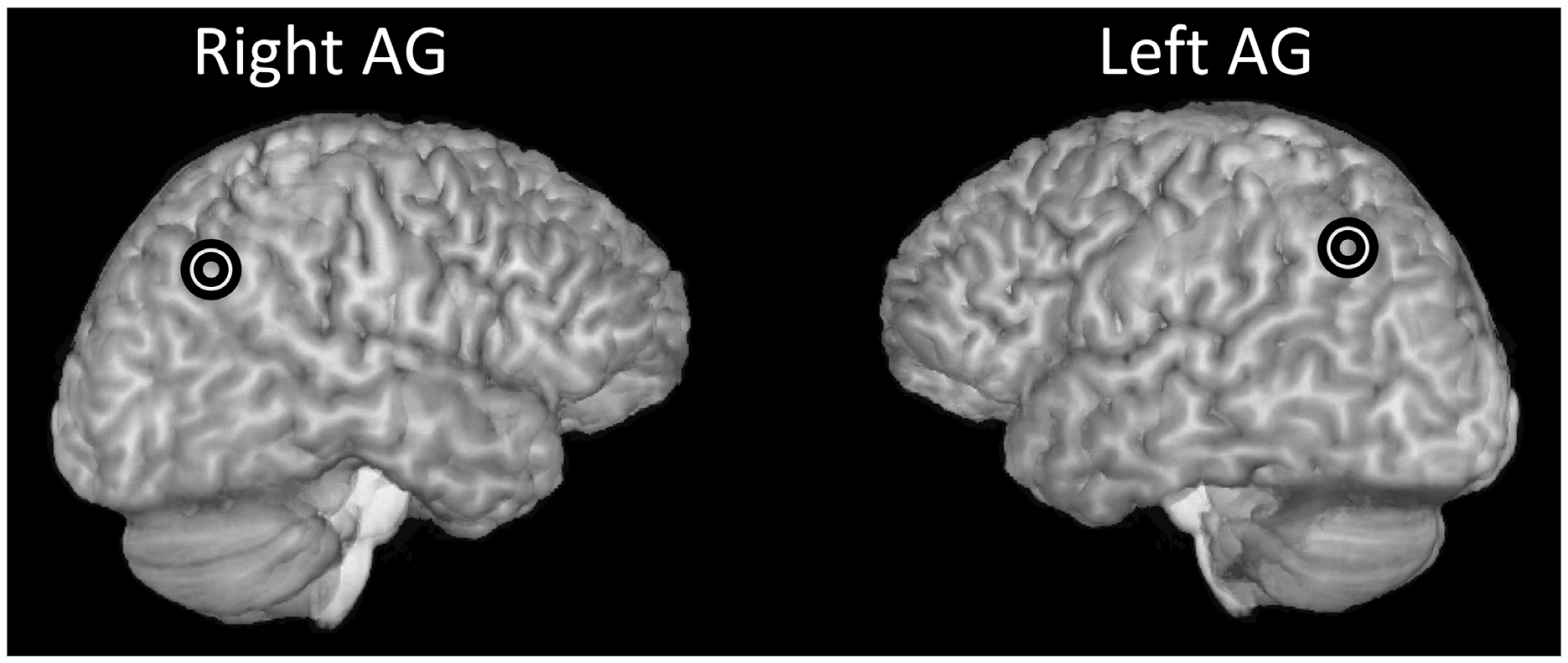
Stimulation sites in the right and left AG, in one participant.

Coordinates for the left hemisphere were the mirror image of the right hemisphere coordinates reported in the three studies. For each participant the MNI brain coordinates from the three studies were converted into native space and then averaged to provide one set of coordinates of the closest cortical surface coordinates. Coil position was then identified by overlaying the scalp with the cortical surface coordinates. The position of the parietal hotspot (see Fig. 1 for an example) corresponded to the anterior bank of the angular gyrus (AG).

### 4. Stimuli Presentation and Experimental Procedure

All participants took part in four experimental sessions carried out at least 24 hours apart. Each session began with a single practice block. In session 1, eight baseline behavioural blocks were completed and no cTBS was administered. In sessions 2, 3 and 4, cTBS was delivered to the left AG (lAG), right AG (rAG) or with the coil in a sham orientation, immediately prior to completing eight test blocks. The order of stimulation conditions in sessions 2–4 was counterbalanced across the sample.

**Figure 2:**
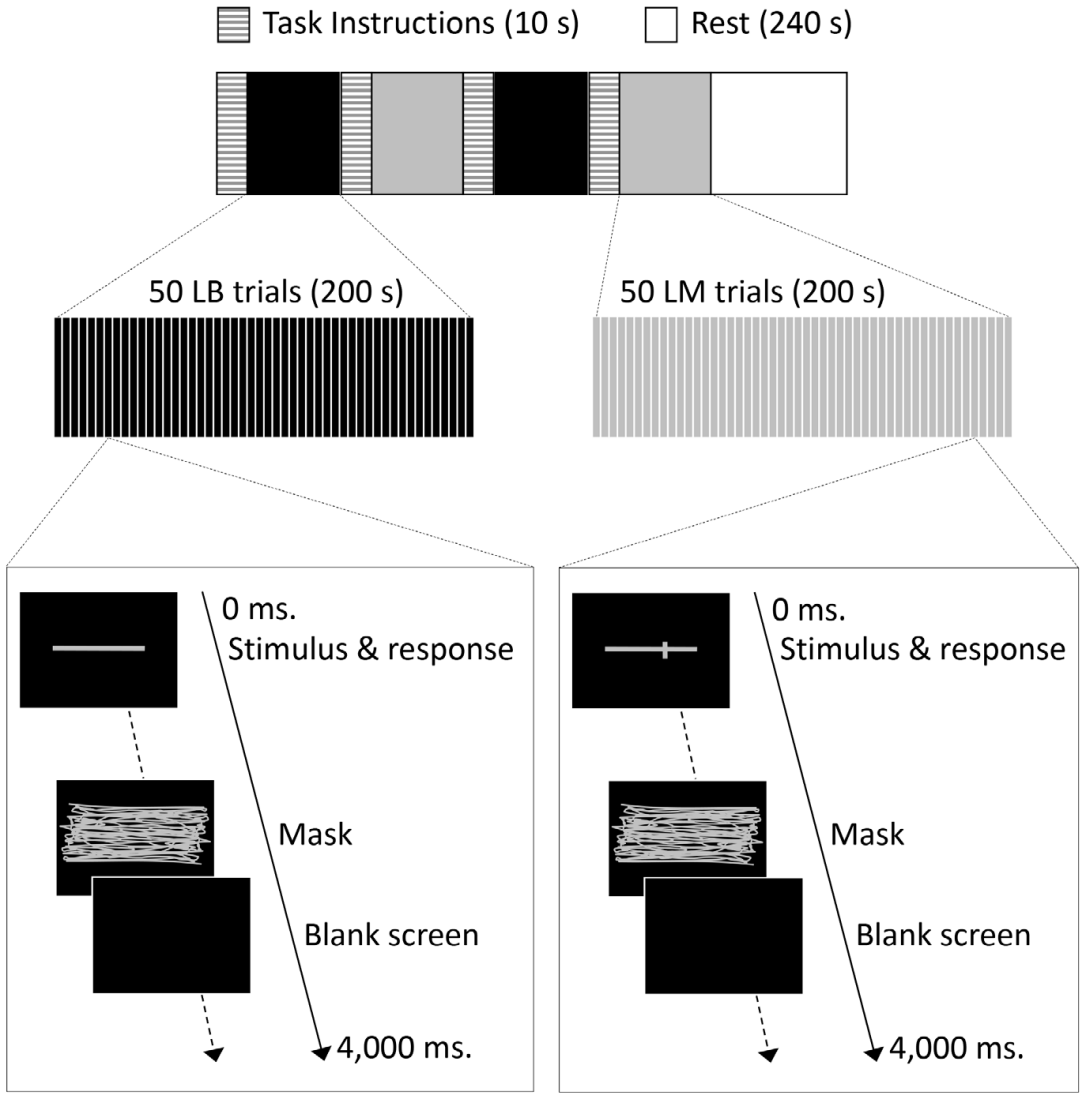
Schematic diagram of the experimental procedure. A single block consisted of task instructions for LB, a set of 50 LB trials, task instructions for LM, and a set of 50 LM trials. Each set of two blocks was separated by a rest period. A single trial consisted of a stimulus, a mask, and a blank screen, which was presented for a variable duration to ensure a fixed overall trial duration.

**Figure 3:**
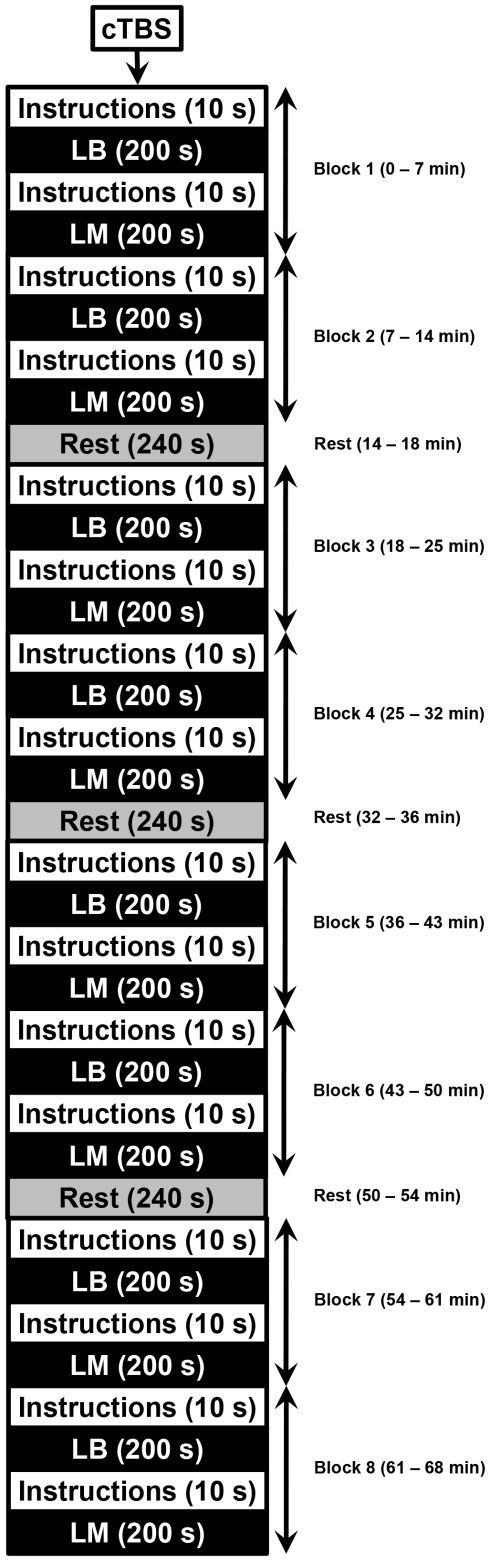
Schematic diagram of the session procedure.

As shown in Figure 2, each single block lasted 420 s and consisted of task instructions for LB (10 s), a set of 50 LB trials (200 s), task instructions for LM (10 s) and a set of 50 LM trials (200 s). After every two blocks there was a 240 s break, allowing the participant to rest. Therefore, a single session that included eight blocks and three rest periods lasted 68 minutes (see Fig. 3 for the session schedule). The order of LB and LM tasks within a single block was the same for all sessions for each participant but counterbalanced across the sample.

Each LB trial consisted of a stimulus line, a mask (500 ms) to abolish retinal after-effects, and then a blank screen. The blank screen was presented for a variable duration to allow for differences in response time, such that the whole trial lasted for a fixed total of 4 s. The line was horizontal and grey on a black background, centred horizontally and vertically on the screen, and subtending a visual angle of 17 degrees (180 mm in length). Participants used a high-speed optical mouse and were instructed to draw a vertical mark (1 pixel in width) at the horizontal midpoint. The starting position of the cursor (a blue dot measuring 1 pixel) was counterbalanced across trials starting within one of the four quadrants of the screen. The dot appeared only when the mouse was moved by the participant and the line was produced only when the participant pressed down continuously on the left mouse button, ending when the participant released the button.

Similar to LB, each LM trial consisted of a stimulus line, a mask and a blank screen. The stimulus was a horizontal grey line (180 mm long, subtending a visual angle of 17 degrees) on a black background, centred horizontally and vertically on the screen, crossed by a small vertical transect (1 pixel wide, 15 mm in height).

Each subset of 50 LM trials consisted of 10 trials each, with transects offset by 4 mm to the left of centre, 2 mm to the left, correctly bisected, 2 mm to the right, or 4 mm to the right. Trials were presented in a random order within each block. Participants were instructed to press one of the three buttons on the mouse to indicate where they considered the vertical transect to be in relation to the centre of the horizontal line, while the stimulus line was on screen; i.e. to the left of centre, to the right of centre or in the centre.

Participants were tested in a dark room, seated in front of a monitor at a fixed distance of 600 mm. The head was rested on a chin-rest, and eye-gaze and pupil diameter were recorded during all blocks using a Cambridge Research Systems 250 Hz Video eye-tracking system. All participants used their dominant right hand to execute responses.

### 5. Scoring and Analysis

In the LB task a vertical bisection mark to the left of centre indicates right pseudoneglect/left bias, bisection to the right of centre indicates left pseudoneglect/right bias and an accurate bisection indicates no pseudoneglect/no bias. A positive score in millimetres indicates a right deviation from centre (left pseudoneglect/right bias) and a negative score indicates a left deviation (right pseudoneglect/left bias). Accurate bisection was scored as zero. Onset times were recorded for critical events: (1) when the stimulus line appeared on screen; (2) when the mouse button was pressed down to start drawing the vertical line; and (3) when the participant had finished drawing their line and the mouse button was released.

In the LM task, responses were assigned a score based on the severity and direction of mis-judgement. A positive score indicates a right deviation (left pseudoneglect/right bias) and a negative score indicates a left deviation (right pseudoneglect/left bias) (see Table 1 for details of the scores assigned). Latencies were recorded from when the stimulus line appeared on screen to when the participant pressed a button to respond.

**Table 1:** LM scores assigned to responses.

Transect Position	Response	Score	Indication
Centre	Right	−1	Moderate right pseudoneglect/left bias
Left	Centre	−1	Moderate right pseudoneglect/left bias
Left	Right	−2	More severe right pseudoneglect/left bias
Centre	Left	1	Moderate left pseudoneglect/right bias
Right	Centre	1	Moderate left pseudoneglect/right bias
Right	Left	2	More severe left pseudoneglect/right bias
Centre	Centre	0	Correct judgement
Left	Left	0	Correct judgement
Right	Right	0	Correct judgement

When the transect was in the centre of the horizontal line but judged by the participant to be right of centre, or when the transect was to the left but judged to be in the centre, a score of −1 was given, indicating a moderate right pseudoneglect/left bias. When the transect was to the left but judged to be to the right, a score of −2 was given, indicating a more severe right pseudoneglect/left bias. When the transect was in the centre but judged to be left of centre, or when the transect was to the right but judged to be in the centre, a score of 1 was given, indicated a moderate left pseudoneglect/right bias. When the transect was to the right but judged to be to the left, a score of 2 was given, indicating a more severe left pseudoneglect/right bias. Correct judgements were scored as zero.

Data following cTBS for both LB and LM included responses from the last seven blocks of trials: the first of the eight blocks was excluded from the analysis to account for the observation that the effects of cTBS on cortical excitability can take approximately 10 minutes to peak [51]. The second block began seven minutes after cTBS was administered; i.e. after one block lasting 420 s. The baseline session for both tasks also included responses from the last seven blocks of trials in order to make the data comparable with sham.

## Results

All data and analyses associated with this article can be downloaded at http://dx.doi.org/10.6084/m9.figshare.701525.

### 1. Classification of Participants as‘Left’ or ‘Right’ Deviants

Participants were separated *a priori* into groups with either a mean left or right deviation (from centre) at baseline, independently on LB (left deviants, *n* = 15; right deviants, *n* = 9) and LM tasks (left deviants, *n* = 12; right deviants, *n* = 12). Outliers were identified independently in each group (left or right deviants; LB or LM) with percentile criteria set to 0.999 and 0.001 (99.9th percentile). To qualify for exclusion, participants were required to be outliers for both sets of sham-normalised data (i.e. lAG minus sham; rAG minus sham). This rule was determined prior to data collection. Two participants were excluded from further analysis on LB and two participants on LM (one participant per bias group, per task).

**Figure 4:**
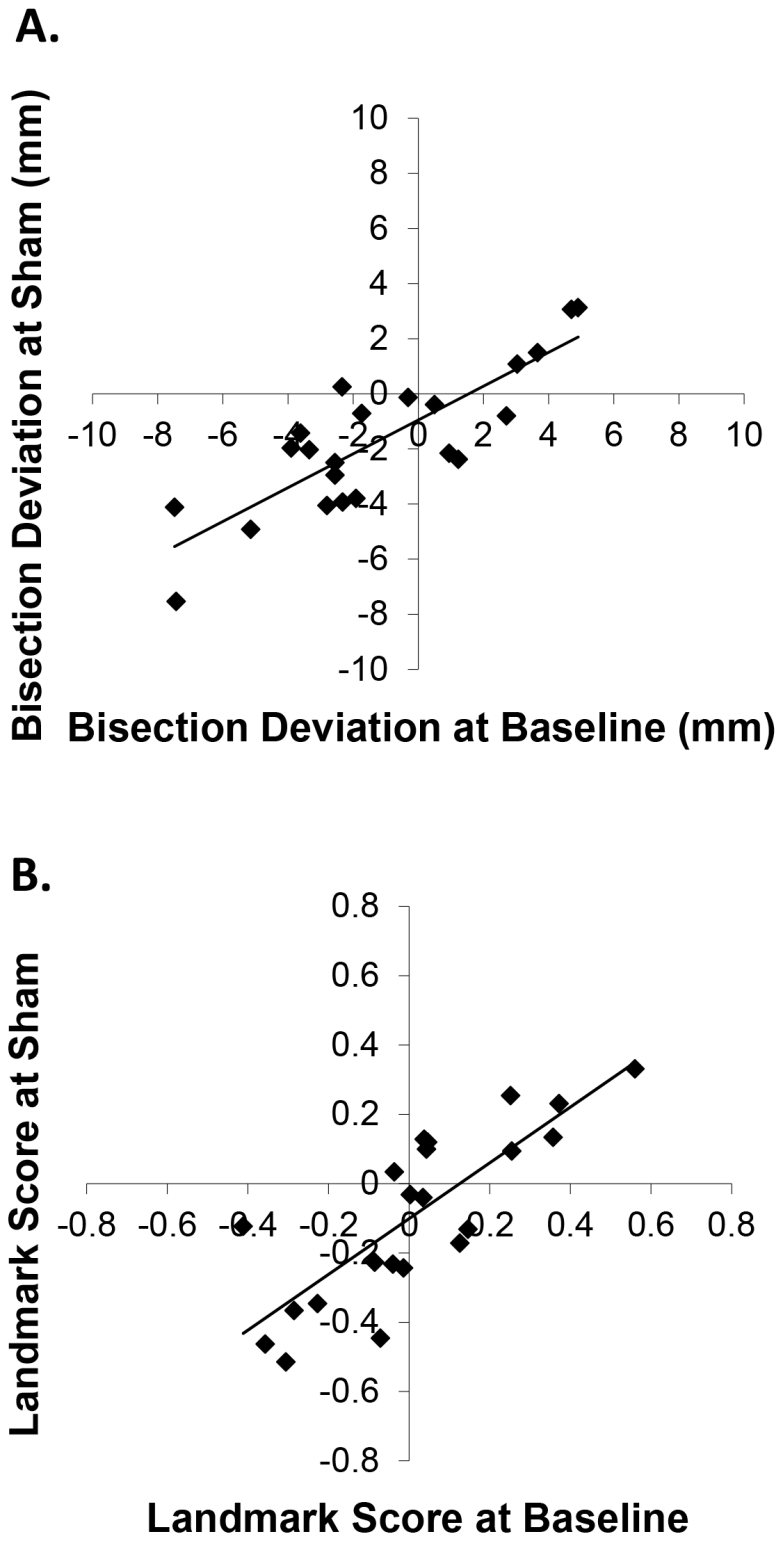
(a) Correlation between LB deviations at baseline and following sham cTBS. (b) Correlation between LM scores at baseline and following sham cTBS.

Baseline data was used to ensure that the participants’ classification as left or right deviants was independent of the cTBS experiment. Sham was used as a control condition in the subsequent analyses, as it matched the session procedures to the active cTBS sessions as closely as possible without stimulating the brain, unlike putative control sites. To confirm that the use of baseline as an independent marker of deviation and sham as the control condition were appropriate, the reliability of deviation was tested. Pearson’s correlations showed that mean baseline LB performance was significantly correlated with sham (Pearson’s *r* = .84, *p*<.001: see Fig. 4a) indicating high retest reliability in bisection deviations. Similarly, mean baseline LM performance was significantly correlated with sham (Pearson’s *r* = .80, *p*<.001: see Fig. 4b) indicating robust retest reliability in bisection judgements.

With regards to LB, while there was a strong correlation between baseline and sham, mean deviations revealed a tendency for both left and right deviants to progress towards zero and become more reliable in the sham condition relative to the initial baseline condition (baseline means: left deviants = −3.39 [SD = 2.05], right deviants = 2.71 [SD = 1.69]; sham means: left deviants = −2.84 [SD = 2.09], right deviants = 0.38 [SD = 2.15]). This could be explained by practice effects in both groups of deviants; i.e., they both become more accurate and reliable. *T*-tests revealed a significant reduction in LB deviations between baseline and sham for right deviants (Cohen’s *d* = 1.20, *p* = .0003; *d* = 0.26, *p* = .22, for left deviants). This cannot be an effect of brain stimulation; rather it reflects practice effects on the bisection task that are more prominent in right deviants. This effect was also revealed by <1 slope (tan) of the correlation line between the sham and the baseline conditions. The slope is formed by both conditions and, therefore, indicates that the practice effect occurred in both conditions. Further investigation revealed that in LB some of the right deviants reversed their bias at sham. Absolute values of LB deviations in the baseline condition, from participants whose deviance reversed, were compared to those that did not reverse. An independent samples *t*-test within the right deviants group revealed that participants who showed a reversal had significant smaller biases in the baseline condition (M = 1.34; SD = 0.96) than those who did not (M = 4.08, SD = 0.88; *d* = 3.43; *t* [6] = −4.2, *p* = .006). In summary, there was a general progression of the mean towards zero across the sample due to performance becoming more accurate and reliable, but rightward deviants had a weaker bias compared to left deviants making them more likely to demonstrate bias reversal. Together, this confirms that the two groups of deviants, as defined at baseline, had been appropriately identified.

Could the effect of right AG cTBS on LB scores in right deviants be related to their overall weaker and less consistent bias? To test this we divided the right deviants into two sub-groups: those whose change in LB score between baseline and sham was greater than the median, and those for whom it was less than the median. In both sub-groups (N = 4 each), right AG cTBS caused a rightward shift in LB scores. In the ‘higher-change’ group the cTBS effect was M = 1.25 mm, SD = 0.94 mm, *d* = 0.69, *p* = .038 via one-tailed *t*-test. In the ‘lower-change’ group, the cTBS effect was M = 0.89, SD = 0.50 mm, *d* = 0.56, *p* = .018 via one-tailed *t*-test. Within right deviants, there was no significant correlation between the magnitude of the cTBS induced effect and the magnitude of the change between baseline and sham (Spearman’s Rho = −0.36, *p* = .39). This correlation was also non-significant in left deviants (Spearman’s Rho = −0.20, *p* = .47). In a separate test, we split the right deviants into those whose LB scores reversed (crossed zero) between baseline and sham and those scores remained right of zero. Again, both groups showed a consistent rightward shift in LB scores following right AG cTBS. In the ‘reversals’ group, the cTBS effect was M = 1.36 mm, SD = 0.85 mm, *d* = 1.05, *p* = .025 via one-tailed *t*-test. In the ‘no-reversals’ group, the cTBS effect was M = 0.78 mm, SD = 0.52 mm, *d* = 0.80, *p* = .028 via one-tailed *t*-test. These analyses provide no evidence to suggest that the increased rate of reversals in right deviants (due to a weaker bias being affected by practice) was related to the effect of right AG cTBS on line bisection.

For the LM task, there was a trend toward a leftward shift in mean deviation scores in left deviants between baseline and sham (baseline mean = −0.18 [SD = 0.25]; sham mean = −0.29 [SD = 0.25]; *d* = 0.44; *p* = 0.09), which reached statistical significance in right deviants (baseline mean = 0.19 [SD = 0.18]; sham mean = 0.09 [SD = 0.15]; *d* = 0.61, *p* = 0.03). Consistent with LB, this difference may reflect a practice effect in right deviants. Some participants reversed their bias at sham so absolute values of their LM scores in the baseline condition were compared to those that did not reverse. An independent samples *t*-test in the right deviants revealed no significant difference between those who did reverse (M = 0.08; SD = 0.07) and those who did not (M = 0.24; SD = 0.19; *d* = 1.09; *t* [10] = 1.6, *p* = 0.13), although the direction of this trend was consistent with the LB task.

### 2. Line Bisection

**Figure 5:**
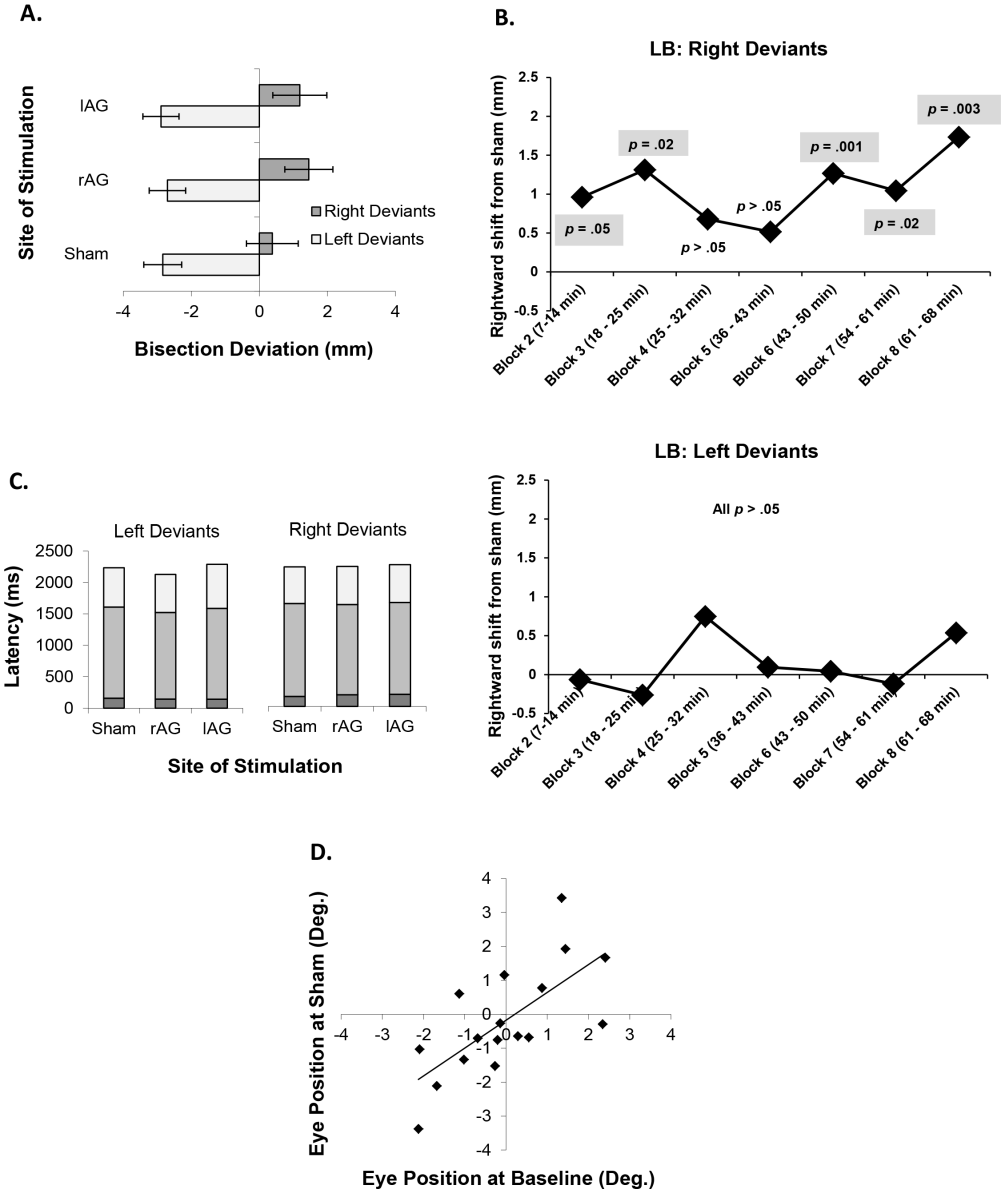
(a) Effect of cTBS on LB deviations in left and right deviants. (b) Time course of the effect of rAG cTBS on LB in right deviants. (c). Effects of cTBS on LB latencies in left and right deviants. Latencies were separated into three time periods: dark grey = the time from the stimulus line onset to when the participant first moved the cursor; mid grey = the time from when the cursor was first moved to when the participant started to draw a bisection mark; light grey = the time from the start to the end of drawing the bisection mark. (d) Correlation in the LB task between eye position at baseline and following sham cTBS. Error bars = ±1 standard error.

To determine the behavioural effects of cTBS, Bonferroni-corrected planned comparisons were performed on the three conditions (sham, lAG and rAG) independently for left and right deviants. Comparisons revealed that cTBS of rAG modulated bisection performance in right deviants, producing a significant rightward shift relative to sham by 1.07 mm [0.1 degrees of visual angle; *d* = 0.52; *t*(7) = −4.18, *p* = .004: see Fig. 5a]. Left deviants, however, were not significantly affected by cTBS of rAG [*d* = 0.07; *t*(13) = −0.49, *p* = .63].

To test whether the effect of rAG stimulation on line bisection (in right deviants) was modulated by time, we undertook an additional two-way ANOVA including factors of cTBS Site (sham, rAG) and cTBS Block (2–8, note that block 1 was excluded from the analysis; see Methods). This analysis revealed a significant main effect of cTBS Site [*F*(1,7) = 17.4, *p* = .004] but no significant main effect of cTBS Block [*F*(6,42) = 1.7, *p* = .15] and no significant cTBS Site by cTBS Block interaction [*F*(6,42) = 1.6, *p* = .18]. Paired *t*-tests between Sham and right AG for each block, revealed significant rightward shifts in five of the seven blocks (*d* for significant effects ranging from 0.53 to 0.76; see Fig. 5b). The effect was reliably significant in the three latest blocks, indicating a persistent TBS effect that is longer than may be predicted by some previous studies (e.g. [55]) but consistent with others (e.g. [51], [52]). The duration of the TBS-induced effect on behaviour is likely to depend on a range of stimulation- and task-specific factors. The precise factors that influence such aftereffects are still poorly understood. However, this analysis confirms that the effects we observed on LB are not diluted by any reduction in this aftereffect. For line bisection in left deviants the two-way ANOVA revealed no main effect of cTBS Site [*F*(1,13) = .24, *p* = .63], no main effect of cTBS Block [*F*(6,78) = .96, *p* = .46] and no significant cTBS Site by cTBS Block interaction [*F*(6,78) = 1.6, *p* = .14]. To test if this null effect was consistent across all blocks we undertook paired *t*-tests between Sham and right AG for each block. All seven tests were non-significant (all *p*>0.08, all *d*<0.38).

Disruption of lAG in left or right deviants did not significantly modulate performance relative to sham (both *p*>.05; both *d*<0.38), although in right deviants there was a trend for a rightward shift relative to sham by 0.81 mm [0.08 degrees; *d* = 0.37 *t*(7) = −1.76, *p* = 0.12]. Analysis of corresponding latencies revealed that cTBS of rAG or cTBS of lAG had no effect on overall response times relative to sham (*p*>.05 and *d*<0.31 for all comparisons) in left or right deviants (see Fig. 5c).

The mean horizontal eye position at baseline was significantly correlated with sham (*r* = .70, *p = *.002; see Fig. 5d) indicating reproducible individual differences in gaze patterns. To determine the relationship between LB performance and eye position, mean Pearson’s *r* values between LB deviations and eye position were calculated for each participant on a trial-by-trial basis and compared to zero. A single-sample *t*-test revealed a significant positive relationship [*M* = 0.24, *SD* = 0.17; *d* = 1.41; *t*(17) = 5.98, *p*<.0001], indicating – as expected – that eye position accounts for a small but reliable proportion of variability in bisection deviations.

Having established a baseline relationship between eye gaze and LB performance, we next tested if the effects of cTBS on LB deviations could be explained by changes in eye gaze. Bonferroni-corrected planned comparisons were performed on the mean eye position during the three conditions (sham, lAG and rAG) independently for left and right deviants. Comparisons revealed that cTBS had no significant effect on eye position (*p*>.05 and *d*<0.25 for all comparisons). However, since some of the eye tracking data was missing (due to poor registration for some subjects) the sample size for right deviants was relatively restricted (*n* = 7; left deviants *n* = 11). Therefore, it remains possible that a significant difference in eye position following cTBS would be revealed in a larger cohort.

The mean pupil diameter at baseline was significantly correlated with sham (*r* = .85, *p*<.0001) indicating high reliability in arousal levels. To determine the relationship between deviations and arousal levels, trial-by-trial within-participant Pearson’s *r* values between LB deviations and pupil diameter were compared to zero. In contrast to the eye position, there was no significant deviation from zero [*M* = −0.01, *SD* = 0.08; *d* = 0.12; *t*(17) = -.53, *p* = .60] indicating that the pupilometric measure of autonomic arousal did not reliably predict LB performance.

### 3. Landmark

**Figure 6:**
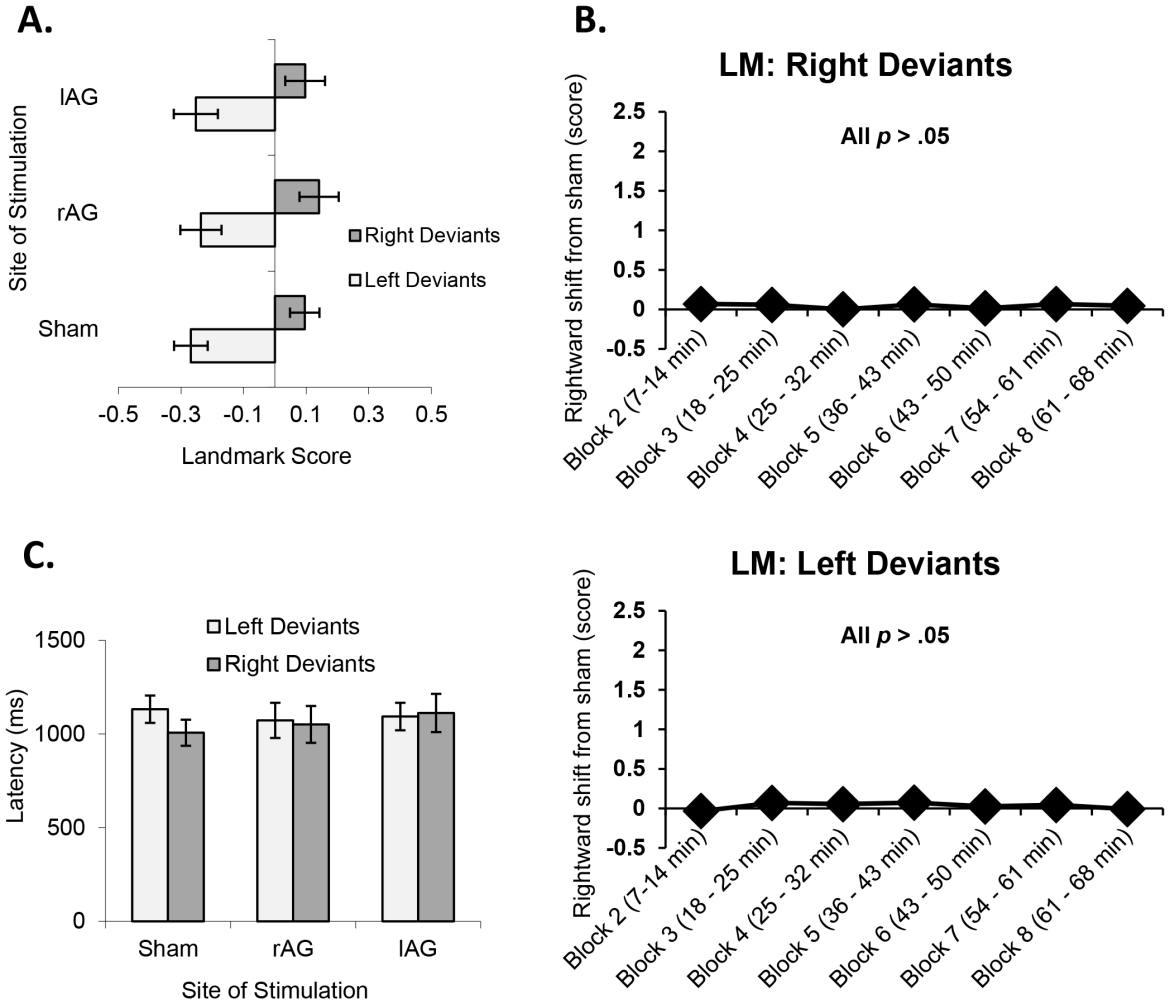
(a) Effect of cTBS on LM scores in left and right deviants. (b) Time course of the effect of rAG cTBS on LB in right and left deviants. (c) Effects of cTBS on LM latencies in left and right deviants. Error bars = ±1 standard error.

To determine the behavioural effects of cTBS, Bonferroni-corrected planned comparisons were once again performed on the three conditions (sham, lAG and rAG) independently for left and right deviants. Comparisons revealed that cTBS had no significant effect on LM performance relative to sham (*p*>.05 and *d*<0.25 for all comparisons; see Fig. 6a). A two-way ANOVA and paired *t*-tests for LM following right AG stimulation, independently for both left and right deviants, was also carried out. There were no significant effects (all *p*>.05; all *d*<0.40; see Fig. 6b), suggesting that the observed behaviour was consistent across time. Analysis of corresponding latencies revealed that cTBS had no significant effect on overall response times relative to sham (*p*>.05 and *d*<0.37 for all comparisons) for left or right deviants (see Fig. 6c).

### 4. Line Bisection vs. Landmark

**Figure 7:**
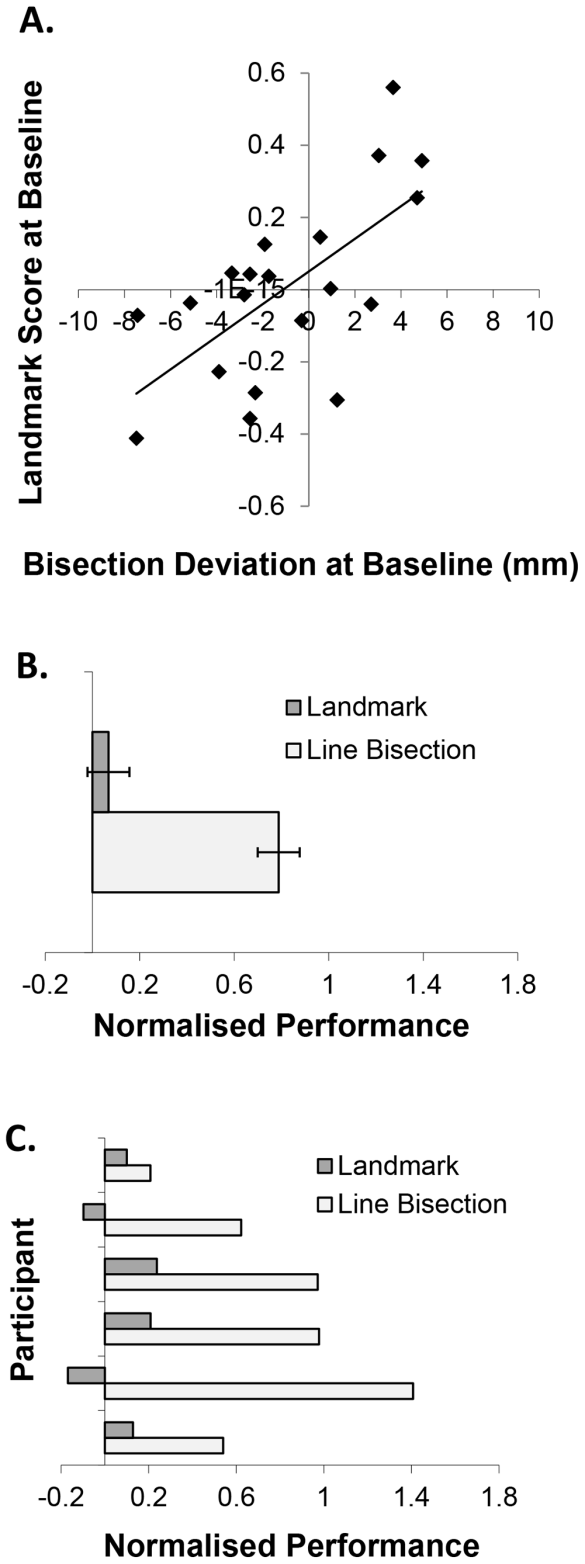
(a) Correlation between LB deviations and LM scores at baseline. (b) Effect of cTBS of rAG on sham-normalised LB and LM performance (i.e. rAG minus sham). (c) Effect of rAG cTBS on LB and LM performance in each ‘right deviant’ subject. Error bars = ±1 standard error.

Mean baseline LB performance was significantly correlated with baseline LM performance (*r* = .65, *p* = .002; see Fig. 7a) indicating a relationship between measures and replicating previous observations [56], [57]. Fourteen of the 20 participants showed the same direction of bias in the LB versus LM.

To determine if there was a quantitative dissociation between the behavioural effects of rAG stimulation on LB versus LM in right deviants, a Wilcoxon Signed Ranks test was applied to normalised data (i.e. rAG minus sham) from participants who were right deviants in both tasks. A significantly greater behavioural effect of rAG cTBS in the LB task versus LM was observed (*z* = 2.02, *n* = 6, *p* = .028; *d* = 2.26; *t*(5) = 3.59, *p* = .016; see Fig. 7b and 7c).

## Discussion

The results of the present study demonstrate the importance of pre-existing (normal) visuospatial asymmetry for informing and predicting patterns of visuospatial neglect. We report evidence that pseudoneglect predicts some of the behavioural effects of disruption to the right parietal cortex. Our results show that having an existing pattern of left pseudoneglect/right bias that resembles actual left neglect renders an individual more likely to exhibit left neglect-like behaviour following cTBS of right AG on the manual LB task. This predictive attribute of the phenomenon provides support for the view that neglect and pseudoneglect arise from a common or linked neural mechanism.

Despite the many studies examining healthy bisection performance, most pay little attention to the substantial variability across healthy individuals and fail to make the distinction between those who consistently demonstrate left pseudoneglect and those who demonstrate right pseudoneglect (although see [42]). These ‘subtypes’ of visuospatial representation may contribute to the behavioural variability observed in patients following right parietal damage.

In the current study, it is important to acknowledge that the effects were found with manual LB but not the perceptual LM task. This distinction between perceptual and perceptual-motor processing has already been established in the bisection literature, with some patients demonstrating neglect on LB but not LM, while others show the reverse [32], [33]. Indeed, Oliveri and Vallar [26] reported rightward errors in healthy participants, symptomatic of left visual neglect, on the non-manual LM task following rTMS of the right supramarginal gyrus but not the AG. Our results are consistent with these findings and suggest that cTBS of right AG does more than simply influence the visual representation of space; it appears to alter the visuo-motor coupling. Furthermore, a purely perceptual account might predict changes in response times and/or eye gaze in either task following cTBS. That is, if the line had been perceived shorter, due to inaccurate representation of the linear extent, it is conceivable that the time it takes to respond to and scan the stimuli should also be shorter; however, this effect was not observed, supporting the idea that stimulated brain region plays a critical role in some, but not all, aspects of visuospatial processing.

There continues to be much debate over the brain area(s) responsible for visual neglect. The area most commonly associated with neglect is the right posterior parietal cortex, particularly the region around the temporoparietal junction [58], [59], [60]; however, neglect has also been observed following more focal strokes of the right inferior parietal cortex, including the AG and supramarginal gyrus [29], [31], [61]. Most recently, evidence has been found for a larger parieto-frontal network in the right than left hemisphere and a significant correlation between the degree of anatomical lateralization and asymmetry of performance on LB [28]. It is clear that neglect cannot be linked to only a single brain area but depends on a widespread network of components and connecting pathways. It is conceivable that this variability and inconsistency may reflect differences in pre-existing patterns of visuospatial cognition and associated differences in neural functioning. For example, individuals who consistently deviate to the right (i.e. left pseudoneglect/right bias) may depend more on their right AG for maintaining the spatial distribution of attention, compared with those who deviate to the left (i.e. right pseudoneglect/left bias).

It is important to note how the results fit in with the model of interhemispheric rivalry; a dominant account of interhemispheric interactions which posited that the hemispheres engage in a ‘see-saw-type’ rivalry [62]. According to this account, each hemisphere attends primarily to the opposite side of space, while inhibiting the capacity of the other hemisphere to do the same. If one hemisphere is damaged or disrupted, the intact side is thought to be released from inhibition, resulting not only in deficient attention for space contralateral to the lesion but also potentially in excessive attention ipsilesionally. In the current study a dissociation between the effect of cTBS to rAG and cTBS to lAG was observed, with only rAG stimulation modulating bisection performance: a dissociation that supports the rivalry account. However, the rivalry account would also predict opposite effects of lAG vs. right AG stimulation: an effect which was not observed in the current study. Rightward deviants demonstrated a similar trend (i.e. a rightward shift) following cTBS to lAG and rAG yet the rivalry account would predict opposite effects. With regards to the null result following lAG, it is possible that a stronger stimulation was needed to elicit an effect, the site location and/or coil orientation was suboptimal, or that lAG itself is not singularly critical for the spatial distribution of attention.

There is evidence that the rivalry account may not be the only possible account for our results. Studies on the somatosensory system [63], the motor system [64], [65], and the visual system [66], [67] have demonstrated that the hemispheres may not always be in direct competition. Rather, it is probable that many varieties of interhemispheric interactions exist [68], including excitatory influences as well as rivalrous, and may depend on the exact task, the brain regions involved and the TMS protocol [63].

In summary, our findings demonstrate a link between healthy cognitive processes and patterns of simulated pathology, and highlight the need to incorporate patterns of normal visuospatial asymmetry into models of healthy spatial cognition and visual neglect. However, it is important to be cautious in our conclusions: it is possible that the effects we observed are specific to cTBS and may not translate to clinical neglect. There are important differences between our observations and those found in patients with visual neglect. For example, LB errors can be considerably larger in patients, right-hemisphere stroke leads to bisection deviation in the majority of patients, and patients can demonstrate significant impairment on LM tasks. At the very least, methodologies of future research on neglect and pseudoneglect should first draw upon the nature of normal spatial cognition to interpret the disorders that follow. Furthermore, it may be possible to exploit these effects clinically [69]; with future studies - perhaps employing cTBS and fMRI – focusing on the differences in the neural underpinnings of pre-existing biases. By understanding how these systems compensate or change following disruption, it may be possible to tailor theory-based rehabilitation strategies that utilize pre-existing biases in visuospatial cognition.
